# Streptavidin functionalized polymer nanodots fabricated by visible light lithography

**DOI:** 10.1186/s12951-015-0084-6

**Published:** 2015-03-28

**Authors:** Clemens Wolfesberger, Richard Wollhofen, Bianca Buchegger, Jaroslaw Jacak, Thomas A Klar

**Affiliations:** Institute of Applied Physics, Johannes Kepler University Linz, Altenberger Str. 69, 4040 Linz, Austria; Department of Medical Engineering, Upper Austria University of Applied Sciences, Campus Linz, Garnisonstr. 21, 4020 Linz, Austria

**Keywords:** Nanolithography, Functional polymers, Fluorescence microscopy, STED-Lithography, Nanodots

## Abstract

**Background:**

Two-photon polymerization, optionally combined with stimulated emission depletion (STED) lithography, allows two and three dimensional polymer fabrication with structure sizes and resolution below the diffraction limit. Structuring of polymers with photons, whose wavelength is within the visible range of the electromagnetic spectrum, gives new opportunities to a large field of applications e.g. in the field of biotechnology and tissue engineering. In order to create new biotechnological applications, versatile methods are needed to functionalize the polymeric structures.

**Results:**

Here we report the creation of polymer-nanodots with high streptavidin (SA) affinity via two-photon polymerization (TPP). Controlling the size of the polymer dots allows for limiting the number of the SA molecules. TPP dots with a diameter of a few 100 nm show up to 100% streptavidin loading. We can show that most of the dots are loaded by one to two streptavidins on average. Attached streptavidin molecules remain functional and are capable to bind 0.7 biotin molecules on average.

**Conclusion:**

The presented functionalized nanostructures may be used as platforms for a multitude of biological experimental setups. Nanoscopic well defined structures, capable of selective binding of streptavin proteins, used as linkers for other biotinylated biomolecules, may also find application in in-vitro sensing, like for example lab on chip devices with limited surface area.

## Background

In 1997, Kawata and his group employed visible light for three-dimensional two-photon-induced lithography [1]. Nowadays, two-photon polymerization (TPP) is used to write features with lateral sizes of 90 [2], 80 [3,4], and 65 nm [5] using pulsed lasers for two-photon excitation with wavelengths of 1030, 800 and 520 nm, respectively.

Recently, STED- and STED-inspired diffraction-unlimited lithography has been realized [6-10]. In STED-lithography [11], one laser pulse excites photoinitiators for radical polymerization and a second laser locally inhibits the ability of the photoinitiator to start the polymerization in the outer rim of the excitation point spread function (PSF). Thereby, polymerization is restricted to the inner part of the PSF, thus creating a shrunken “effective PSF”. Such a STED-based approach allows the generation of structures with feature sizes below the limits of TPP. Currently, feature sizes as small as 55 nm and a resolution of 120 nm can be achieved [12].

Mixing of different acrylate precursors allows for tuning the polymer properties such as mechanical stiffness, surface charge and surface energy which in turn can tune the degree of hydrophobicity or improve protein adhesion [13,14]. Applying STED–lithography, we were able to show that single antibodies can be attached to quasi 0-dimensional polymer dots, the so called ‘nanoanchors’ [15]. The engineering of protein nanoarrays is of great importance for medical and biological applications, including biosensors and drug screening. Protein nanopatterns with tunable features can for example be used to mimic an in vivo-like environment in order to precisely manipulate the behavior of living cells to analyze the interaction between the cells and matrix [16-20].

**Figure 1:**
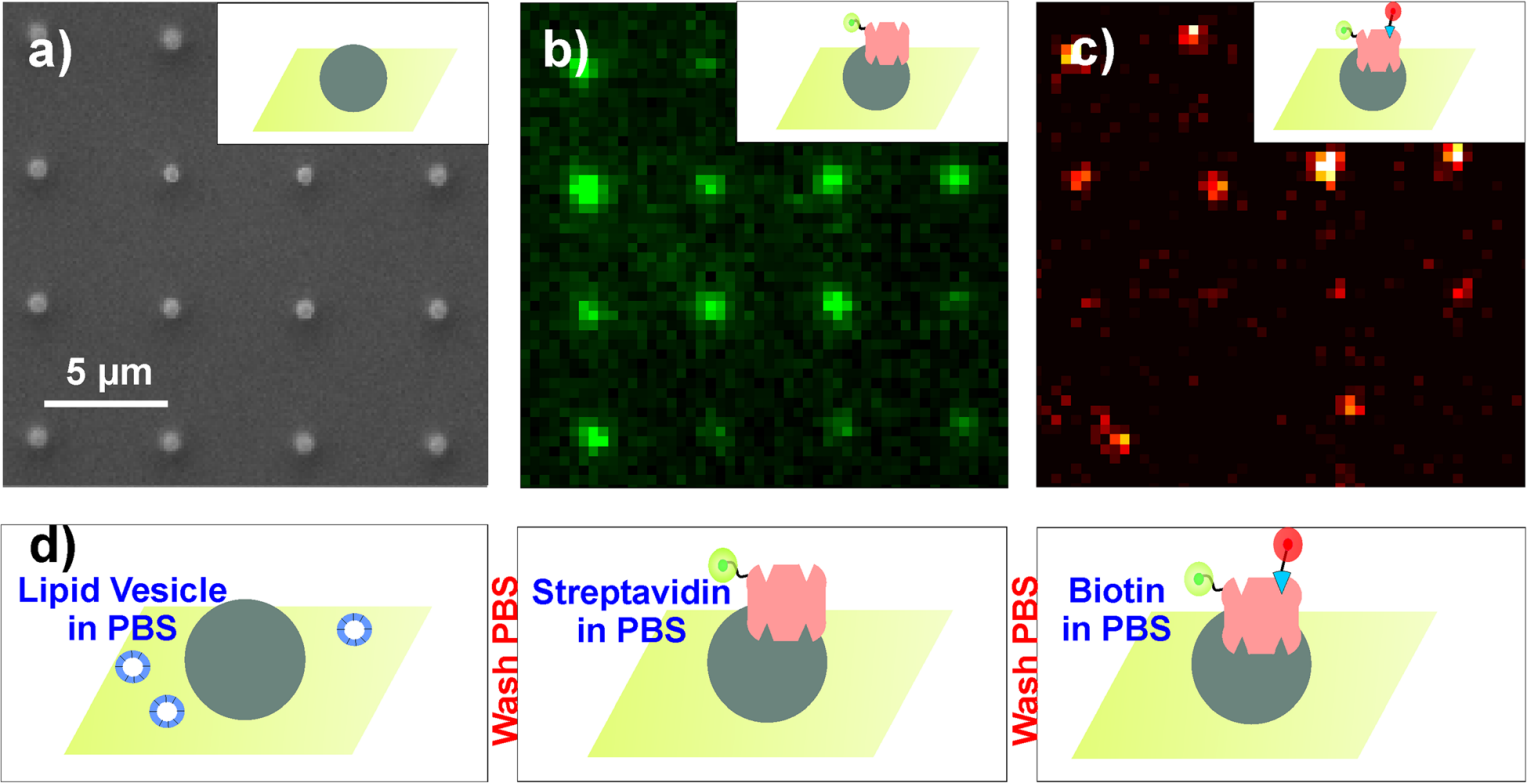
**Incubation of nanodots. a)** SEM image of TPP fabricated nanodots with a surface area of 1×10^5^-2×10^5^ nm^2^, polymerized with Irgacure 819 photoinitiator. **b)** Nanodots incubated with Alexa555-streptavidin (@ 532 nm excitation). **c)** Nanodots with streptavidin after additional Atto655-biotin incubation (@647 nm excitation). All images were taken with 10 ms illumination time, no Irgacure 819 autofluorescence was detected for such a short time interval. **d)** A sketch of the incubation process for the nanodots. First phosphate buffered saline (PBS) solution with lipid vesicles is added. After the incubation, the slide is washed with PBS and Alexa555-streptavidin dilution is added. Subsequently after an additional PBS washing step, we add Atto655-biotin diluted in PBS.

The goal of this work was to establish a highly versatile method for conjugating nanoscopic polymer structures with any desired molecule. Due to the high protein affinity of the structured polymer [15], it is well suited for the use of the streptavidin/biotin crosslinking system, a commonly used protein-based linker system for coupling of molecules [20-24]. The streptavidin/biotin binding belongs to the strongest non-covalent interactions (K_s_ ~ 10^13^ M^−1^) known in biology [21]. Biotin-associated hetero-bifunctional linkers with reactive chemical groups or even macromolecules are widely accessible [25]. In order to characterize the streptavidin adhesion to two-photon nanostructured dots, we varied the dot size, the substrate properties and the concentration of fluorescently labeled streptavidin and biotin during incubation. Figure 1a shows a zoomed scanning electron microscopy (SEM) image of a TPP fabricated nanodot array. In a second step, the dots are loaded (Figure 1d) with streptavidin (labeled with Alexa555 in this case, Figure 1b). Subsequently we add biotin which specifically binds to streptavidin (labeled with Atto655, Figure 1c).

## Results

### Lithography of polymer nanodots

Structured nanodots of various sizes were fabricated using TPP or STED-lithography. Polymer ‘nanoanchors’ with sizes as small as 65 nm in diameter can be achieved using STED–lithography [15]. The TPP fabricated dots described in this paper are up to ~300 nm in diameter.

**Figure 2:**
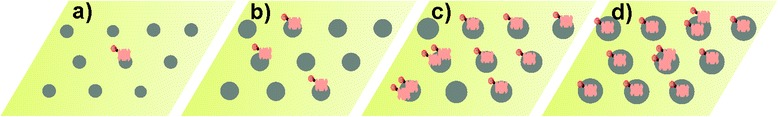
**Schematic drawing of nanostructured dots. a)** Sketch of nanostructured dots (nanoanchors) fabricated via STED-lithography and incubated with fluorescent streptavidin. Only 10% of all dots were covered with proteins. **b)** Sketch of TPP fabricated dots having ~30% average streptavidin loading per dot. **c)** and **d)** are sketches of the largest fabricated dots with over 80% and 100% streptavidin coverage.

We use a (80/20) mixture of the two acrylate monomers SR499 (Sartomer, Colombes Cedex, France) and pentaerythritol triacrylate (PETA, Sigma-Aldrich, Vienna, Austria) including 300-400 ppm monomethyl ether hydroquinone with either 0.25 wt% 7-diethylamino-3-thenoylcoumarin (DETC, Acros Organics, Geel, Belgium) as a depletable photoinitiator [26] for STED-TPP or Irgacure 819 (BASF, Ludwigshafen, Germany), an efficient and nonfluorescent photosensitive initiator for ordinary TPP. This photoresist was shown in a previous study to have antibody affinity [15]. For fabrication of nanodots with a diameter of ~70 nm (surface areas < 10^4^ nm^2^), we used STED-lithography with 5.5 mW excitation and 28.8 mW depletion power. Without depletion beam, two-photon excitation powers between 5.5 and 6.5 mW are applied to create nanodots with surface areas of 1-2 × 10^4^ nm^2^, 1-2 × 10^5^ nm^2^ and > 2 × 10^5^ nm^2^. Hence, dots of four different sizes are written. They are arranged in arrays of 30 × 30 or 40 × 40 dots with 2 μm dot spacing each. Figure 2 shows a schematic drawing of four arrays of nanodots with various sizes and various streptavidin loading. The smallest dots, shown in Figure 2a, are fabricated with STED-lithography and show the lowest loading with streptavidin (~10% only, *vide infra*). TPP fabricated dots have a size dependent streptavidin loading reaching up to 100% for the largest dots (sketched in Figure 2d).

The quality of the arrays is analyzed via SEM (Figure 1a) and fluorescence microscopy using an excitation wavelength of 492 nm. The STED-lithography fabricated dots contain DETC and are hence visible due to their intrinsic fluorescence with an emission maximum at 525 nm. Irgacure 819 exhibits only a very weak broadband fluorescence when excited with 492 nm and 532 nm light. Depending on the photo-initiator, we can either use red fluorophores for labeling (Atto655 (Atto-tec GmbH, Siegen, Deutschland)) on the DETC containing STED-lithography fabricated dots, or use green and red fluorophores for labeling (e.g. Alexa555 and Atto655) when TPP with Irgacure 819 is performed. The low autofluorescence of Irgacure 819 does not disturb the quantitative signal analysis.

The streptavidin is either labeled with Atto655 or Alexa555 fluorophores (see Materials: Labeling of Streptavidin). For incubation, the streptavidin is dissolved in phosphate buffered saline (PBS) (Sigma-Aldrich, Vienna, Austria) and dropcast on to the samples. To avoid unspecific streptavidin binding to the substrate, we use two different coating strategies, either 5000-PEG-silane or a supported lipid bilayer [27] for glass surface passivation. For the first strategy, we coat the surface with PEG, which is known to reduce protein adsorption due to its hydrophilic nature [28,29], prior to writing the polymer dots. For the second strategy, the dots are written on a bare glass and subsequently, a bilayer coating is formed by spreading of palmitoyloleoylphosphatidylcholine (POPC) lipid vesicles [30]. The main advantage of lipid passivation is the short incubation time, high bilayer homogeneity and the ability of self-recovery. However, bilayer formation only works in physiological buffers. In comparison, passivation with PEG is less dependent on environmental factors but more vulnerable to surface defects [31,32].

Fluorescence images are taken after incubation of the STED-lithographically fabricated nanoanchors or the TPP dot arrays with Atto655 or Alexa555 labeled streptavidin, respectively. We performed a stepwise increase of the amount of streptavidin (2 μl of the stock solution per 5 min incubation-step each) until the nanodots are saturated.

**Figure 3:**
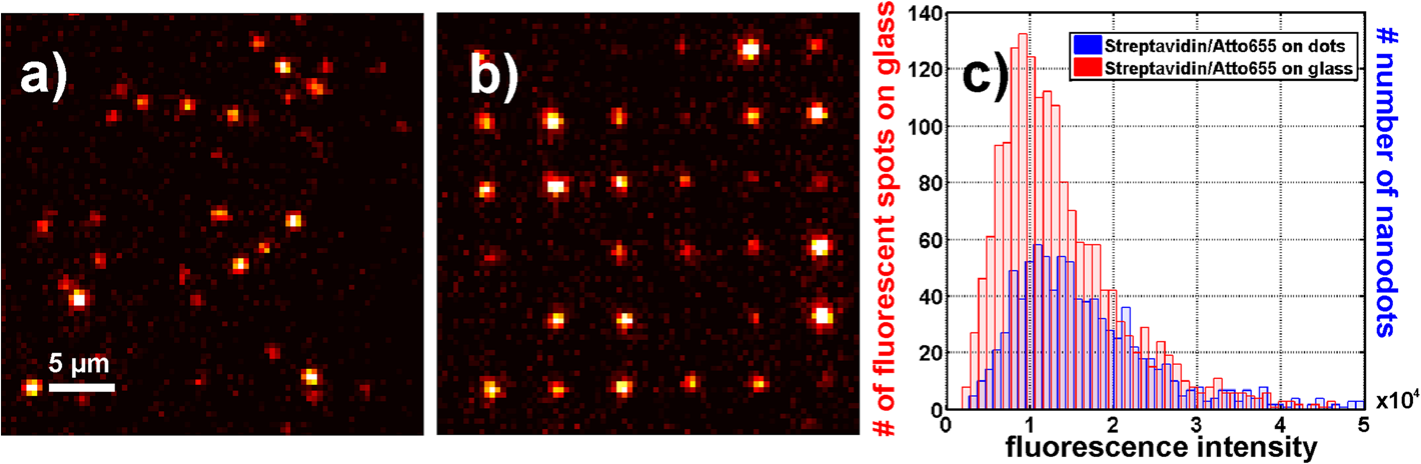
**Intensity distributions of Atto655-Streptavidin single molecules.** Comparison of the single molecule Atto655-streptavidin signal on glass and on 1 × 10^5^-2 × 10^5^ nm^2^ nanodots. **a)** Image of sparsely and randomly distributed Atto655-labeled streptavidin on a substrate without acrylic patches passivated by lipids at 647 nm excitation (unwashed). **b)** Fluorescence after incubation of the TPP nanodots with Atto655-labeled streptavidin, after washing (647 nm excitation). Over 80% of all dots carry at least one streptavidin. **c)** Statistical distribution of fluorescence intensity counts per fluorescent spot during 10 ms illumination time, obtained from: (red) sparsely and randomly distributed streptavidin as shown in **a)** and (blue) from nanodots loaded with streptavidin (see **b**).

Figure 3a shows an image of Atto655 labeled streptavidin loosely attached to a lipid-coated glass slide. Since a lipid supported bilayer exhibits strong repulsive forces against proteins [27], we used high concentrations of streptavidin without washing of the sample to introduce at least some unspecific binding. High probability to find single streptavidin on the lipid coated glass is used to quantify the fluorescence strength of single streptavidin in order to achieve a statistic of single molecule emission strength shown in Figure 3c (red histogram).

Single molecule fluorescence microscopy is used to analyze the streptavidin loading of the polymer nanodots. For an estimation of the loading rates, the fluorescence signal from occupied nanodots (Figure 3b) is compared with signals of single streptavidin molecules sparsely distributed on lipid passivated glass (Figure 3a). To quantify the fluorescence signals of the single streptavidin molecules on glass as well as streptavidin incubated nanodots, isotropic undecimated wavelet transformation [33] is used for the recognition of individual fluorescent spots [34]. Further parameterization of the fluorescence signals is performed by Gaussian fitting.

The Atto655 labeled streptavidin molecules which were bound to the nanodots are shown in Figure 3b. Due to the PEG passivation, almost no unspecific binding of the streptavidin is observed after washing. The distribution of fluorescence counts from nanodots loaded with streptavidin is shown in Figure 3c (blue histogram, > 500 single molecule signals). The intensity distributions of streptavidin on lipid passivated glass (red) and on nanodots (blue) differ, which indicates that quite a number of dots have more than one SA molecule attached.

### Streptavidin loading of nanodots

We find that only ~10% of all STED-lithographically fabricated nanoanchors with ~70 nm average diameter (surface areas <10^4^ nm^2^) are loaded with at least one streptavidin. This result was rather unexpected since we were able to load 98% of STED-lithographically fabricated nanoanchors with antibodies in a previous study [15]; i.e. the affinity of proteins to nanoanchors seems to strongly depend on the specific type of protein. Since only ~10% of all patches were loaded by streptavidin on average, the STED-lithographically fabricated nanoanchors were not considered for further analysis.

In order to achieve a more detailed characterization of the streptavidin binding properties, TPP nanodots with different surface areas are polymerized. We classified the dot area into three groups; the first groups surface area is ~2 × 10^4^ nm^2^, the second group covers around 1 × 10^5^-2 × 10^5^ nm^2^, and the third category comprises areas above 2 × 10^5^ nm^2^. The dot area was approximated by a spheroid which was parameterized by atomic force microscopy. These area classes correspond roughly to dots with diameters of <150 nm, 200–300 nm and slightly >300 nm, respectively. The nanodot surface is not only given by the diameter of the nanodot, but also by the nanodots height. This height can be adjusted by positioning the excitation point spread function axially with ~15 nm precision.

**Figure 4:**
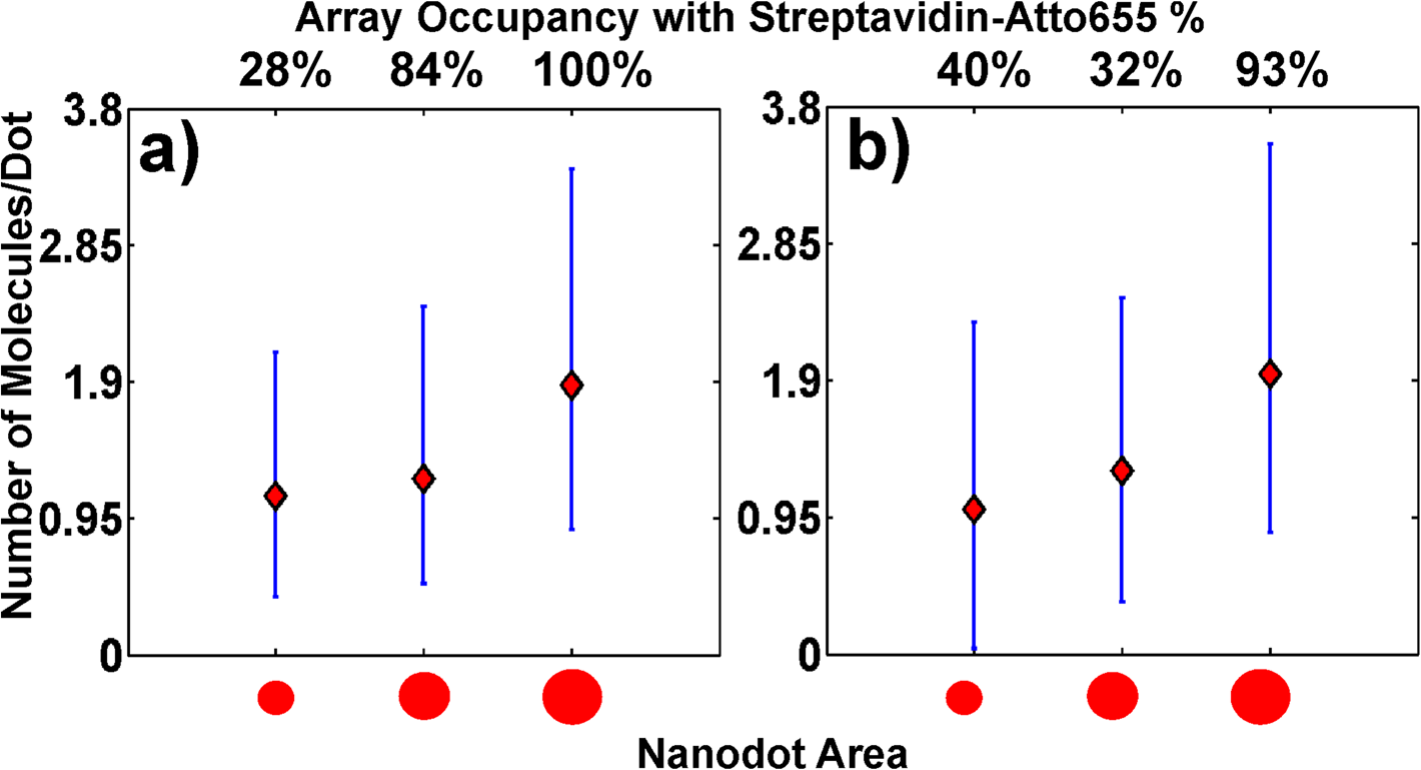
**Average nanodot Atto655-Streptavidin loads with respect to dot surface area.** Above the graphs: The probability that a nanodot carries at least one Atto655-SA. Graphs: Average number of Atto655-SA per nanodot, which carry at least one Atto655-SA for surface areas <2 × 10^4^ nm^2^, 1 × 10^5^-2 × 10^5^ nm^2^ and > 2 × 10^5^ nm^2^. Graphs **a)** and **b)** are for differently passivated substrates: **a)** lipid passivation, **b)** PEG passivation.

Figure 4 shows the loading of TPP nanodots with Atto655-streptavidin with respect to the three nanodot surface areas and for lipid (Figure 4a) or PEG (Figure 4b) passivated sample surfaces. For instance, in case of the PEG passivated TPP nanodots with surface areas of <2 × 10^4^ nm^2^, only 40 ± 9% of all dots carry at least one streptavidin molecule. These 40 ± 9% of SA carrying dots carry on average one streptavidin complex (1.3 = σ^+^; 0.96 = σ^−^). In case of the lipid bilayer passivated surface, 28±9% of all measured dots are labeled with an average of 1.1 streptavidin/dot (1 = σ^+^; 0.7 = σ^−^).

For PEG passivated arrays with larger TPP dots (i.e. 1 × 10^5^-2 × 10^5^ nm^2^ dot surface area), 32 ± 11.6% of all TPP dots carry 1.27 streptavidin complexes (1.2 = σ^+^; 0.91 = σ^−^). When the substrate is passivated with lipids, a much better total dot occupancy of 84 ± 22.4% is achieved with 1.22 streptavidin molecules per labeled dot (1.2 = σ^+^; 0.73 = σ^−^). The improved streptavidin coverage of the dots on the lipid coated slides is not fully understood. An explanation may be the difference in the thickness of the two passivation layers. The POPC lipid bilayer has usually a thickness of 5–6 nm [35], whereas 5000-PEG molecule form a ~27 nm (if fully stretched [36]) high ‘mesh’ if exposed to an aqueous solution [28,37]. Hence, due to the adhesion of the PEG backbone to the nanodot, the PEG layer may screen a part of the TPP nanodot surface from proteins [28,37].

For the largest TPP dot arrays of >2×10^5^ nm^2^ surface area, fabricated on a PEGylated surface, we observe that 70-93% of all dots are occupied with an average of 1.94 streptavidin molecules (1.6 = σ^+^; 1.1 = σ^−^). In case of the lipid bilayer passivated substrate with >2×10^5^ nm^2^ dot surface area, 100% of all measured dots are loaded with at least one streptavidin and 1.87 streptavidins (1.51 = σ^+^; 1 = σ^−^) on average. In order to verify the influence of the fluorophores on the streptavidin binding properties, intermediately sized TPP dots with 1×10^5^-2×10^5^ nm^2^ area are incubated with Alexa555-streptavidin. The Alexa555-streptavidin incubated arrays, fabricated on PEGylated slides, have 36 ± 7% of all dots loaded with at least one streptavidin with an average of 1.2 streptavidin per loaded dot (1.36 = σ^+^; 1 = σ^−^). In case of lipid passivated substrates, 53 ± 13% of all dots are occupied by at least one SA and these occupied dots carry 1.3 streptavidin molecules on average (1.35 the upper error; 1 the lower error).

### Binding of biotin

To prove whether the streptavidin is still biochemically active, the TPP dots were incubated with a fluorophore–biotin conjugate. For these experiments we used Atto655 labeled biotin and Alexa555 labeled streptavidin. Figure 1 depicts a section of a nanodot array with an average dot surface area between 1×10^5^ and 2×10^5^ nm^2^, sequentially incubated with Alexa555-streptavidin and Atto655-biotin (Figure 1b, c). We determined an average binding of 0.7 biotin molecules per Alexa555-conjugated streptavidin. This result clearly illustrates that binding pocket accessibility is influenced by the adhesion to the nanodot, since a native streptavidin possesses four biotin binding pockets [21]. However, we find that on TPP dots with 200–300 nm in diameter, statistically at least one biotin molecule is attached.

## Discussion

STED-lithography and TPP offer the possibility of generating nanostructures, which allow a versatile protein coating. Recently, we were able to show that STED-lithographically fabricated polymer nanoanchors with properly adjusted chemical properties have the ability to bind a single antibody [15]. To extend the versatility of the polymer structures, streptavidin has been used for coating this time. Streptavidin is frequently used as a coupling agent because of its high affinity to biotin [21,23]. Nowadays, biotinylated macromolecules of almost any type are commercially available, rendering streptavidin/biotin a well-suited system for polymer functionalization.

Surprisingly, the adhesion of the streptavidin to the polymer (80/20 SR499 and PETA mix) is significantly weaker compared to antibody adhesion. Only ~10% of all STED-lithographically prepared nanoanchors were occupied by at least a single streptavidin as compared to 98% in the case of antibodies [15]. The value is independent from the substrate passivation either by PEG or lipids.

To achieve higher streptavidin loading, larger TPP fabricated nanodots with surface areas <2 × 10^4^ nm^2^, 1 × 10^5^-2 × 10^5^ nm^2^ and >2 × 10^5^ nm^2^ were tested. We observed that on the nanodots with surface areas <2 × 10^4^ nm^2^, 28 ± 9% of all TPP nanodots on a lipid passivated substrate were carrying 1.1 (1 = σ^+^; 0.7 = σ^−^) streptavidin on average and 40 ± 9% of the nanodots on a PEG passivated surface array were occupied by 1 (1.3 = σ^+^; 0.96 = σ^−^) streptavidin on average. For TPP dots with slightly larger surface areas of 1 × 10^5^-2 × 10^5^ nm^2^, the number of bound streptavidin remained almost the same, 1.27 (1.2 = σ^+^; 0.91 = σ^−^) and 1.22 (1.2 = σ^+^; 0.73 = σ^−^) molecules per loaded nanodot for PEG and lipid passivated slides, respectively. The occupation efficiency of TPP nanodots changed significantly for lipid passivated slides reaching 84 ± 22.4%, while on PEG slides only 32 ± 11.6% of the dots were occupied. In the case of the biggest structures >2×10^5^ nm^2^, all TPP dots on a lipid passivated arrays carry 1.87 streptavidin (1.51 = σ^+^; 1.1 = σ^−^) on average. 70-93% of nanodot arrays on PEGylated slides are occupied with 1.94 (1.6 = σ^+^; 1 = σ^−^) molecules/dot.

To test the biochemical activity of the streptavidin complexes, the SA functionalized structures were incubated with biotinylated fluorophores. We find that the nanodot bound streptavidin still possesses biotin affinity, although not all four binding pockets are accessible. On average, 0.7 binding pockets per streptavidin are occupied by a biotin molecule.

## Conclusions

We demonstrated that almost a full coverage of TPP fabricated nanodot arrays with one to two streptavidin molecules can be achieved, despite the fact that the streptavidin affinity to SR499/PETA acrylic nanodots is lower than the affinity of antibodies. Furthermore, the preserved biochemical activity of the streptavidin enables a variety of biotinylated macromolecules to be attached. A lower streptavidin adhesion to the polymers limits the dynamic range of the system. Preferably, surfaces with areas >1 × 10^5^ nm^2^ are required to facilitate a homogenous streptavidin coating.

We have shown that TPP fabricated acrylate structures can be functionalized with streptavidin, down to a single molecule level. The straightforward method can be safely applied to functionalize any arbitrarily shaped nanostructure. The adhered streptavidins partially keep biotin binding affinity. Single molecule fluorescence studies proved that on average 0.7 biotin molecules were bound to an attached streptavidin. Nanoscopic well-defined structures capable of selective binding of any possible protein via strepavidin-biotin coupling may find application in *in vitro* sensing such as lab on chip devices with a well-controlled surface area.

## Methods

### Passivation of glass slides with polyethyleneglycol (PEG)

PEG and lipid coated slides are used to minimize unspecific protein adsorption on the glass surface. The PEG-coated slides are passivated according to Pollheimer et al. [38].

First, glass slides are cleaned with Piranha solution (H_2_SO_4_/H_2_O_2_ (7:3)) and subsequently incubated for 20-24 h with a reaction solution containing mPEG-Silane (MW 5000) (Nanocs, New York, USA), anhydrous toluene (10 mg/ml) and 0.08% HCl. Subsequently, the slides are rinsed with ethanol and acetone. The slides are stored for a maximum of one week to exclude possible aging processes of the PEG coated glass slides.

### Passivation of glass slides with lipid bilayers

Palmitoyloleoylphosphatidylcholine (POPC) (Avanti Polar Lipids Inc., Alabama, USA) - Lipid vesicles were produced according to Huppa et al. [30] and kindly provided to us by the Institute of Applied Physics at the Vienna University of Technology. After applying 100 μl phosphate buffered saline (PBS) buffer to the chamber of the lipid slides, 10 μl lipid is added for 15 minutes allowing the lipid to coat the surface. Then, the sample is rinsed with buffer to remove free lipid vesicles.

### Labeling of streptavidin with Alexa555

Atto655 labeled streptavidin was purchased from AttoTec GmbH (Siegen, Germany). Alexa 555 labeled SA was prepared in our own lab as follows: Streptavidin was labeled via N-hydroxysuccinimid (NHS)-ester conjugate Alexa555 (Lifetechnologies, Vienna, Austria) according to protocols provided by AttoTec GmbH (Siegen, Germany). For the labeling, 30 μl of a 1 mg∕ml streptavidin (dissolved in PBS buffer) stock solution were adjusted to a pH of 8.6 with a 0.2-M sodium bicarbonate buffer. A labeling ratio of 1:3 streptavidin to fluorophore has been adjusted. The reaction mixture was incubated for 1 h at room temperature. The labeling solution was purified via a PD-10 Sephadex® G-25 m (GE Healthcare, Pittsburgh) purification column in PBS buffer. To concentrate the labeled streptavidin, the solution was transferred to a cut off filter (3–10.000 MWCO (Molecular Weight Cut Off) PES) and centrifuged at 1200 rpm in a centrifuge (Thermo, Multifuge 1S-R, HERAEUS, Thermo Scientific, Vienna, Austria) until only 100–200 μl remained. The concentrated solution was split into aliquots and stored at −20°C.

### Incubation

PEG–coated slides were allowed to swell in PBS buffer for 15 minutes to improve the surface passivation against proteins and subsequently incubated with labeled streptavidin for 5 minutes. We performed a stepwise increase of the streptavidin amount (2 μL each) until the nanodots were saturated (5 min/incubation-step). After each incubation step, the chamber is rinsed with PBS to remove unbound molecules. The same incubation procedure has been used for lipid coated slides.

### Fluorescence analysis

In order to quantify the distribution of the fluorescence strength of single streptavidin or the nano-anchors functionalized with streptavidin, we applied an isotropic undecimated wavelet transformation [33], used for the recognition of individual fluorescent spots [34], combined with Gaussian fitting for their parameterization [39-41]. Similar analyses were performed for single streptavidin proteins as well as for streptavidin coated polymer dots. To quantify the number of molecules on the dots, the fluorescence signal of each dot has been divided by an average signal of fluorescent labeled streptavidin. The average loading of the dots with streptavidin molecules is then displayed in a histogram.

### TPP/STED-lithography-setup

The two-photon polymerization starters are excited with ultra-short laser pulses (82 MHz repetition rate, 110 fs, 780 nm, FFS-tSHG, Toptica, Gräfelfing, Germany) and become locally depleted in the outer rim of the point spread function by a depletion beam (532 nm, continuous wave, Verdi-V5, Coherent, Santa Clara, CA, USA). The 532 nm depletion beam was shaped into a donut form using a 2π spiral phase mask (RPC Photonics, Rochester, NY, USA) and a λ/4 plate converting the depletion beam into a circularly polarized beam with a handedness that matches the 2π spiral phase plate. A combination of a two axes piezo stage (PI M686.D64, Physikinstrumente, Karlsruhe, Germany) with 25 × 25 mm range, a position accuracy of 0.1 μm (0.3 μm repeatability) on both axes and a three axes piezo stage (PI 562.3CD, Physikinstrumente, Karlsruhe, Germany) with 200x200x200 μm range and a position accuracy of 1 nm (x/y/z-position repeatability of 2/2/4 nm) are used for positioning. An avalanche photo diode (APD) is used for detection of the backscattered light, enabling fine adjustment of the relative focus positions. The setup is controlled with a custom-made LabView®-based software.

### Fluorescence-microscopy-setup

The images were taken on a modified Olympus IX81 (Olympus Austria GmbH Vienna, Austria) inverted microscope. The samples were illuminated through an Olympus UApo N 100× ∕ 1.49 NA oil objective lens (Olympus Austria GmbH Vienna, Austria) with two diode lasers at 642 nm (Omicron Laserage Laserprodukte GmbH—Phoxx® 642, Rodgau-Dudenhofen, Germany), 532 nm (Cobolt Samba 100™, Solna, Sweden) and 491 nm wavelength (Cobolt Calypso 100™, Solna, Sweden). The signal acquisition was carried out on an Andor iXonEM + 897 (back-illuminated) EMCCD (16-μm pixel size) (Andor Technology Ltd., Belfast, UK). The experiments were performed using excitation powers of 0.126 kW∕cm^2^ and 0.025 kW∕cm^2^ at 642/532 nm, respectively. The samples were illuminated for 10 ms (642/532 nm) with 40 ms delay time. A motorized XY-stage is used for the sample movement (SCAN, Märzhäuser Wetzlar GmbH & Co. KG, Wetzlar, Germany). The illumination protocols were timed with a custom-made LabView®-based control software.

## Abbreviations

STED: Stimulated emission depletion
TPP: Two-photon polymerization
PSF: Point spread function
SEM: Scanning electron microscopy
PBS: Phosphate buffered saline
SA: Streptavidin
PETA: Pentaerythritol triacrylate
DETC: 7-diethylamino-3-thenoylcoumarin
PEG: Polyethyleneglycol
POPC: Palmitoyloleoylphosphatidylcholine
MWCO: Molecular weight cut off
APD: Avalanche photo diode
